# Combination therapy analysis of ezetimibe and statins in Chinese patients with acute coronary syndrome and type 2 diabetes

**DOI:** 10.1186/s12944-015-0004-7

**Published:** 2015-02-18

**Authors:** Lulu Li, Minli Zhang, Fuxiang Su, Yang Li, Yali Shen, Jie Shen, Daqing Zhang

**Affiliations:** Department of Cardiology, Shengjing Hospital of China Medical University, Shenyang, China; Charite University of Berlin, Berlin, Germany

**Keywords:** Acute coronary syndrome, Diabetes, Statins, Ezetimibe, Combination therapy

## Abstract

**Background:**

Dyslipidemia management situation in Chinese patients with high risk and very high risk has been demonstrated very low, despite the wide use of statins. The effects and safety of the combined treatment of ezetimibe (EZ) and statins in Chinese patients with acute coronary syndrome (ACS) and type 2 diabetes mellitus (T2DM) remain unknown.

**Methods:**

Chinese Patients with ACS and T2DM were divided into the statins group (*n* = 40) and the combination group of EZ and statins (*n* = 44). In order to evaluate the clinical effects on lipids-lowering, systemic inflammation response and clinical safety, the follow-up of all patients was carried out at day 7^th^ and 30^th^ after treatment.

**Results:**

The level of low-density lipoprotein cholesterol (LDL-C) in combination group and statins group was 1.87 ± 0.42 and 2.18 ± 0.58 mmol/L at day 7^th^, 1.51 ± 0.29 and 1.94 ± 0.49 mmol/L at day 30^th^, respectively. The control rates of LDL-C level in the combination group and the statins group were 77% and 45% at day 30^th^, respectively. There was no significant improvement on high-density lipoprotein cholesterol (HDL-C) level during follow-up. The triglyceride (TG) levels were significantly reduced in both groups, while no obvious difference was observed between two groups. No significant difference on serum high-sensitivity C-reactive protein (hs-CRP) level between two groups was observed. Moreover, we did not observe any significant correlation between serum lipids levels and serum hs-CRP level during follow-up. The liver dysfunction and muscle related side effects (MRSE), creatine kinase (CK) and myopathy were not observed in both groups.

**Conclusion:**

Our study demonstrated that it is feasible to initiate combination therapy during acute phase for Chinese patients with ACS and T2DM, which can bring more significant effect on LDL-C-lowering and improve the control rate of LDL-C level with good safety.

## Introduction

Diabetes mellitus (DM) has been demonstrated as coronary heart disease (CHD) risk equivalent and is associated with 2- to 4-fold increase in the risk of CHD [1]. The risk of acute coronary events or cardiovascular death events of diabetic patients within 10 years is equivalent to the patients with old myocardial infarction. Moreover, diabetes with prior myocardial infarction had much higher cardiovascular mortality (45%) than non diabetes with prior myocardial infarction (MI) (18.8%) [2]. Thus, the risk of cardiovascular events is very high in the acute coronary syndrome (ACS) patients complicated with type 2 diabetes mellitus (T2DM). Dyslipidemia, especially increased low-density lipoprotein cholesterol (LDL-C) level is a pathogenic risk factor for CHD. Meta-analysis showed that the 1 mmol/L reduction in LDL-C bring 23% reduction in cardiovascular disease (CVD) within 5 years [3]. The 2014 AHA/ACC guideline for the management of patients with non-ST-elevation acute coronary syndrome (NSTE-ACS) emphasized that NSTE-ACS patients could benefit more from high-intensity statins therapy in reducing the rate of recurrent MI, CHD mortality and the need for revascularization and stroke [4]. Recently, the DYSIS-China Survey of dyslipidemia management situation in patients treated with lipid-lowering agents showed that the LDL-C control rate in high risk and very high risk population were 54.8% and 39.7%, respectively [5]. Clinical research revealed that the doubling dosage of statins can only bring 6% incremental reduction of LDL-C level, and probably increased the risk of DM and worsen the glycemic control in diabetic patients [6]. Moreover, HPS2-THRIVE study demonstrated that Chinese individuals have much higher risk of myopathy with Simvastatin 40 mg alone (0.13%/year vs. 0.04%/year, p = 0.001) or combined with niacin (0.53%/year vs. 0.03%/year), compared with those of European [7]. Therefore, most of Chinese patients are not tolerable to the intensive dosage of statins as guideline suggested [4]. We need to explore an appropriate cholesterol-lowering therapy for high risk patients in China. Ezetimibe (EZ) inhibits cholesterol absorption from the small intestine by selectively inhibiting the cholesterol transporter, Niemann-Pick C1 like1 (NPC1L1) protein which result in the decreased cholesterol delivery to liver, thus lower the plasma cholesterol level [8]. Statins combined with EZ can further decrease LDL-C levels by 6% ~ 25% in hypercholesterolemia patients who cannot achieve the recommended LDL-C level with statins monotherapy and significantly improve the control rate of LDL-C level [9]. Recent report showed that EZ 10 mg/d combined with low dose pravastatin 10 mg/d can lead to the improvement in insulin resistance (IR) compared with high dose pravastatin 40 mg/d in CHD patients with hyperlipidemia [10], which probably related to the EZ inhibition on the reactive oxygen species production and insulin-induced endoplasmic reticulum stress [11]. Thereby, the patients of metabolic syndrome and T2DM may get additional benefit from the combination therapy of EZ and statins. Nevertheless, the evidence of EZ combined with statins is limited in Chinese CVD with very high risk population. The present study aims to observe the efficacy and safety of the combined lipid-lowering therapy in Chinese patients with ACS and T2DM during acute phase.

## Materials and methods

### Patients

Patients admitted for ACS and T2DM in the Department of Cardiology of Shengjing hospital from December 2011 to December 2012 were enrolled. Patients presented with an ACS including ST-elevation myocardial infarction (STEMI) in 24 h or non-ST-elevation myocardial infarction (NSTEMI) in 48 h and high-risk unstable angina (UA) in 48 h were eligible for inclusion. Hospital ethical boards approved the study.

Exclusion criteria included the chronic inflammatory disease or the history of acute infection within 1 month; connective tissue disease and autoimmune disease which diagnosed clearly; the tumors and aneurysm; the history of operation or injury within 3 months; severe congestive heart failure (New York Heart Association class III-IV) or cardiogenic shock; malignant arrhythmia (atrioventricular block aboveIIgrade, ventricular tachycardia, ventricular fibrillation); received probucol or other lipid-lowering treatment such as nicotinic acid, bile-acid sequestrating, fish oil and garlic products within 8 weeks; impaired hepatic function; the estimated glomerular filtration rate (eGFR) < 30 ml/min; 3 times of the upper limit of normal (ULN) in the creatine kinase (CK) level and no relation with myocardial infarction; thyroid dysfunction. The study was approved by the Ethics Committee of Shengjing Hospital of China Medical University (Shenyang, China). All procedures were performed in accordance with ethical standards. All subjects participated in the study after making signed informed consents.

### Study design

In this prospective, observational and real word study, eligible patients received the statins (rosuvastain 10 mg/d, atorvastatin 20 mg/d simvastatin 20 mg/d and pravastatin 20 mg/d) or combination of EZ 10 mg/d with the above dosage of statins. In addition, all patients were treated with 100 mg/d of aspirin, 75 mg/d of clopidogrel and in appropriate individuals with the treatment of β-blocker, angiotensin concerting enzyme inhibitor or angiotensin receptor blocker, percutaneous transluminal coronary angioplasty and coronary stent implantation.

### Laboratory assays

The plasma lipids and serum hs-CRP were measured from fasting blood samples at admission, at day 7^th^ and 30^th^ after treatment. The primary endpoints include LDL-C level, its control rate and the safety; the secondary endpoint is serum hs-CRP level. The plasma total cholesterol (TC) and triglyceride (TG) were tested by oxidase methods, the plasma high-density lipoprotein cholesterol (HDL-C) was measured by chemical modified enzyme method, the plasma LDL-C was tested by selective dissolution method, and the serum hs-CRP was measured by immune transmission turbidimetric method. The baseline levels of CK, CK-MB, and aspartate aminotransferase (AST) came from the lowest levels within 3 days after admission, considering that the increase of CK, isoenzyme of CK-MB and AST due to myocardial infarction probably interfere with analysis of myopathy and liver dysfunction.

### Safety assessment

Safety evaluation based on patient report, investigator observation and laboratory test during the follow-up. Laboratory safety variables such as alanine aminotransferase (ALT) level that was more than 3 times the ULN or the AST level that was more than 5 times the ULN were considered elevated. Muscle related side effects (MRSE) were defined as CK greater than 5 times the ULN with or without myopathy symptoms.

### Statistical analysis

Data are performed with SPSS 19.0 software and presented as mean ± SE. The paired t test is used to compare the baseline and posttreatment at 7^th^ and 30^th^ in each group. Independent-samples t test is used for comparison between two treatment groups. *χ*^2^ analysis is used to examine the categorical data between treatment groups. P values less than 0.05 are regarded as statistically difference. The relations between lipids levels and serum hs-CRP are tested by bivariate analysis.

## Results

### Baseline characteristics

Of the 157 patients enrolled for the study, 84 patients were matched the inclusion criteria without exclusion criteria. All the 84 patients completed the study. Among these patients, 40 patients received the statins monotherapy (29 on rosuvastatin 10 mg/d, 8 on atorvastatin 20 mg/d, 2 on simvastatin 20 mg/d, 1 on pravastatin 20 mg/d) and 44 patients received the 10 mg/d EZ combined with statins (25 on rosuvastatin 10 mg/d, 15 on atorvastatin 20 mg/d, 2 on simvastatin 20 mg/d, 2 on pravastatin 20 mg/d). The treatment groups were well matched with regard to the baseline characteristics. The average age was 63 years and women accounted for 57%. Of the patients who were received treatment, 54% UA, 28% NSTEMI and 18% STEMI (Table 1).

**Table 1:** **Baseline data of study participants**

**Item**	**Combination group (** ***n*** **=44)**	**Statins group (** ***n*** **=40)**	***P***
Male/female	24/20	24/16	0.614
UA/NSTEMI/STEMI	21/14/9	24/10/6	0.528
ROS/ATOR/SIM/PRA	25/15/2/2	29/8/2/1	0.962
Age (year)	62 ± 1.5	64± 1.5	0.514
BMI	25.1± 0.15	24.7± 0.19	0.136
smoking/non-smoking	18/26	16/24	0.932
TC (mmol/L)	4.70± 0.17	4.43± 0.15	0.252
TG (mmol/L)	1.93± 0.17	2.02± 0.19	0.459
HDL-C (mmol/L)	1.00± 0.03	1.00± 0.04	0.966
LDL-C (mmol/L)	3.07± 0.14	2.70± 0.13	0.053
hs-CRP (mg/L)	4.55± 0.70	4.15±0.80	0.711
ALT (IU/L)	28± 3.5	30± 2.9	0.726
AST (IU/L)	61± 16	52±11	0.658
TBIL (Umol/L)	10.2± 0.68	9.7±0.66	0.724
Cr (μmol/L)	77.5± 4.52	78.6±5.59	0.871
FBG (mmol/L)	7.66± 0.39	8.27±0.62	0.092
HbA1C (%)	7.4±0.32	7.9±0.28	0.187
CK (U/L)	101±29	117±24	0.433
CK-MB (U/L)	23±7	19±6	0.547
TnI (μg/L)	16.17±4.85	12.14±4.52	0.545

Data are presented as mean ±SE. P value less than 0.05 for significant difference between two groups at baseline.

### Levels of the lipids

**Figure 1:**
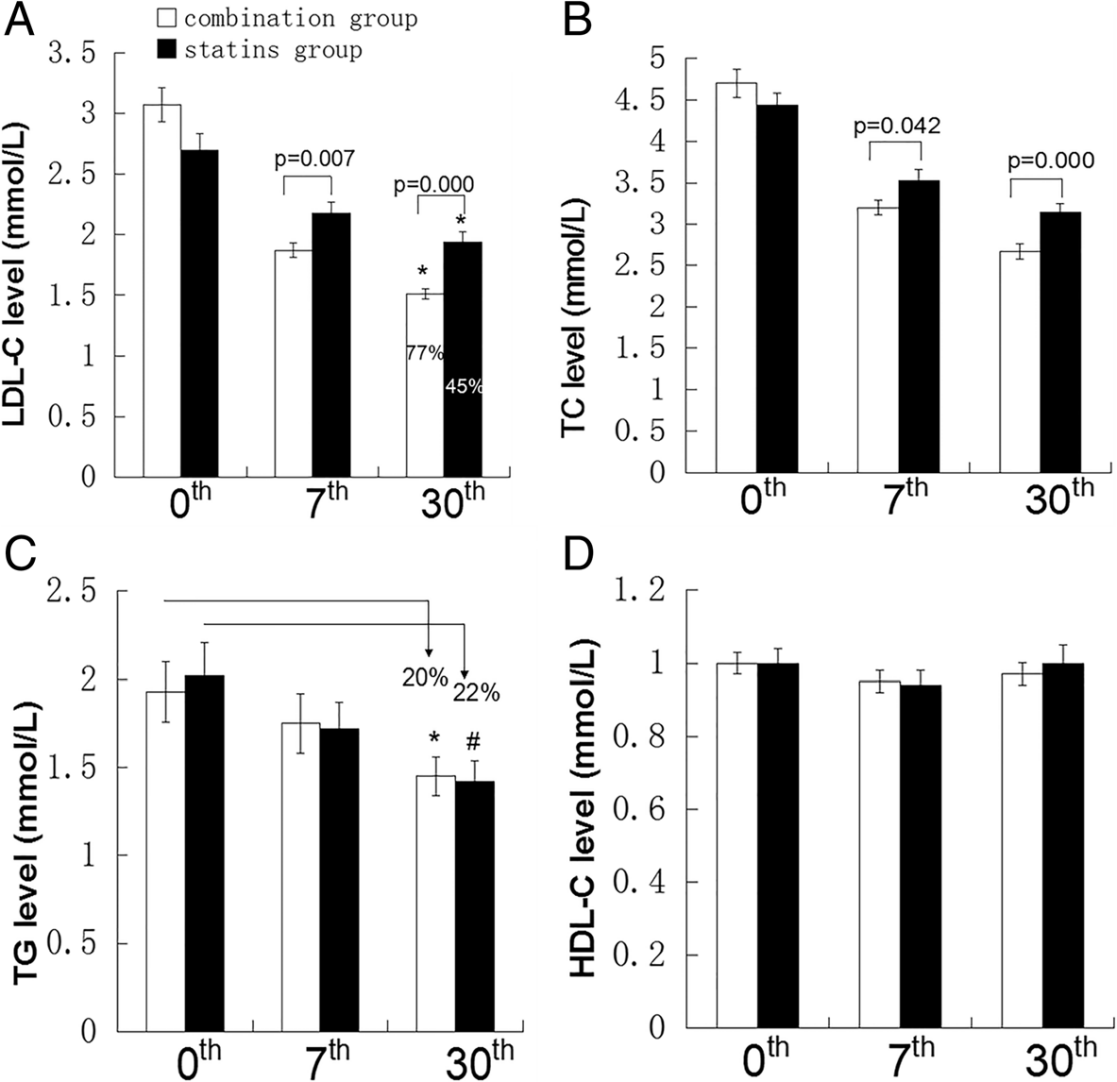
**Plasma lipids levels in both groups during follow-up.** ACS patients with T2DM divided into two groups, the combination group who received EZ 10 mg/d with standard dose of statins (n=44) and statins group who received standard dose of statins (n=40). Plasma lipids levels were tested at baseline, 7^th^ day and 30^th^ day after treatment. **(A)** LDL-C levels and control rate of LDL-C. *Indicate the percentage control rate of LDL-C level, P=0.002. **(B, C, D)** TC, TG, HDL-C levels. *p=0.000 versus TG level at baseline in combination group.#p=0.000 versus TG level at baseline in statins group. Data are shown as mean ± SE. Independent t test was to compare the significant difference of treatment groups. Data expressed as as mean ± SE.

In the present study, compared with statins group, the greater reduction of LDL-C level in combination group was observed at day 7^th^ (39% vs. 18.5%, P = 0.007) and this effectiveness continued through-out the 30 day treatment period (50% vs. 29%, P < 0.001). According to the 2011 ESC/EAS Guidelines for the management of dyslipidemia, the optimal level or relative reduction ratio of the LDL-C level should be less than 1.8 mmol/L or greater than 50% reduction, the greater proportion of patients in the combination group achieved the LDL-C goal attainment, compared with the statins group (77% vs. 45%, P = 0.002) (Figure 1A).

TC levels in two groups were obviously reduced. The TC levels in combination group and statins group were reduced by 42% and 25% at day 30^th^, respectively (Figure 1B).

TG levels in the combination group and statins group were significantly reduced by 20% (p = 0.000) and 22% (p = 0.000) at day 30^th^, respectively. However, no significant difference was observed between two groups (Figure 1C).

Moreover, HDL-C levels in both groups did not show any significant elevation during the 30 days follow-up (Figure 1D).

### Hs-CRP level

**Figure 2:**
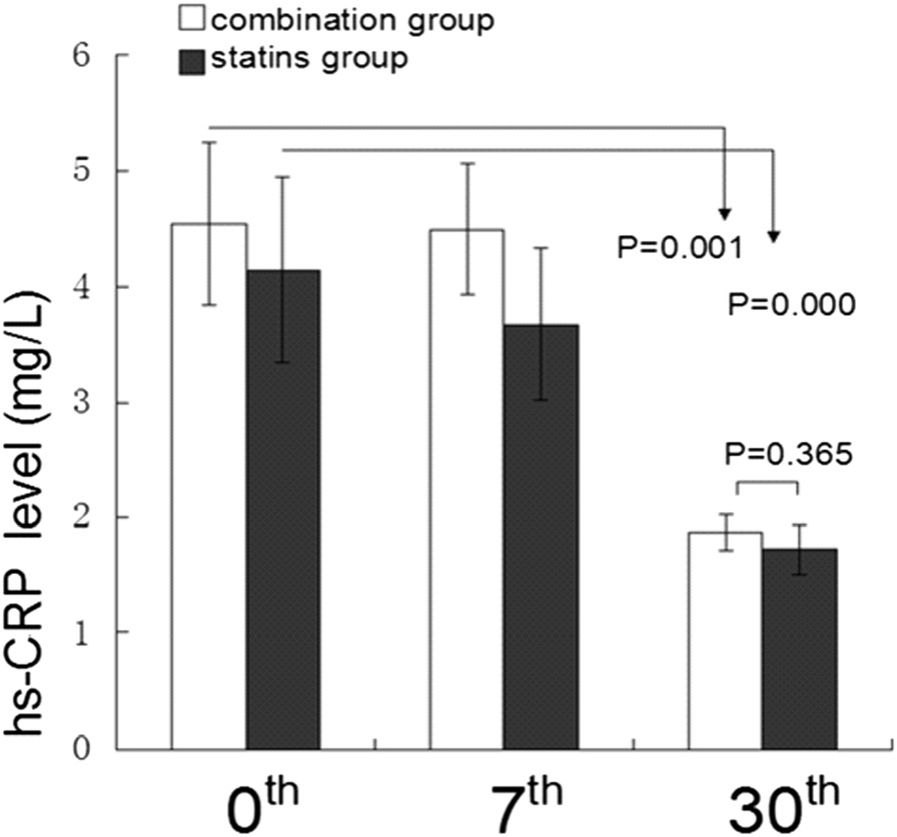
**Inflammation markers of both groups during follow-up.** The anti-inflammatory effects of EZ combined with statins and statins monotherapy were evaluated during follow-up. Hs-CRP level was measured at baseline, 7^th^ day and 30^th^ day. Paired t test was used to compare significance of pre and posttreatment. Independent t test was used to examine the difference between the treatment groups. Data are expressed as mean ± SE.

In addition to the lipid-lowering effects, we also observed that serum hs-CRP levels were obviously decreased in the combination group by 47% (P = 0.000) and statins group by 42% (P = 0.001) at day 30^th^, compared with the baseline levels. Nevertheless, there was no significant difference on hs-CRP reduction between two groups (P = 0.365) (Figure 2).

Furthermore, we tested the relationship between serum hs-CRP and plasma lipids. Bivariate analysis showed that there was no significant correlation between serum hs-CRP level and plasma lipids levels (Table 2).

**Table 2:** **Correlation between hs-CRP and lipids levels**

		**TC**	**LDL-C**	**TG**	**HDL-C**
Hs-CRP	R	-0.242	-0.135	-0.291	0.210
	P	0.133	0.068	0.140	0.193

Data are presented as mean ±SE. P value are less than 0.05 for significant correlation.

### Safety evaluation

**Figure 3:**
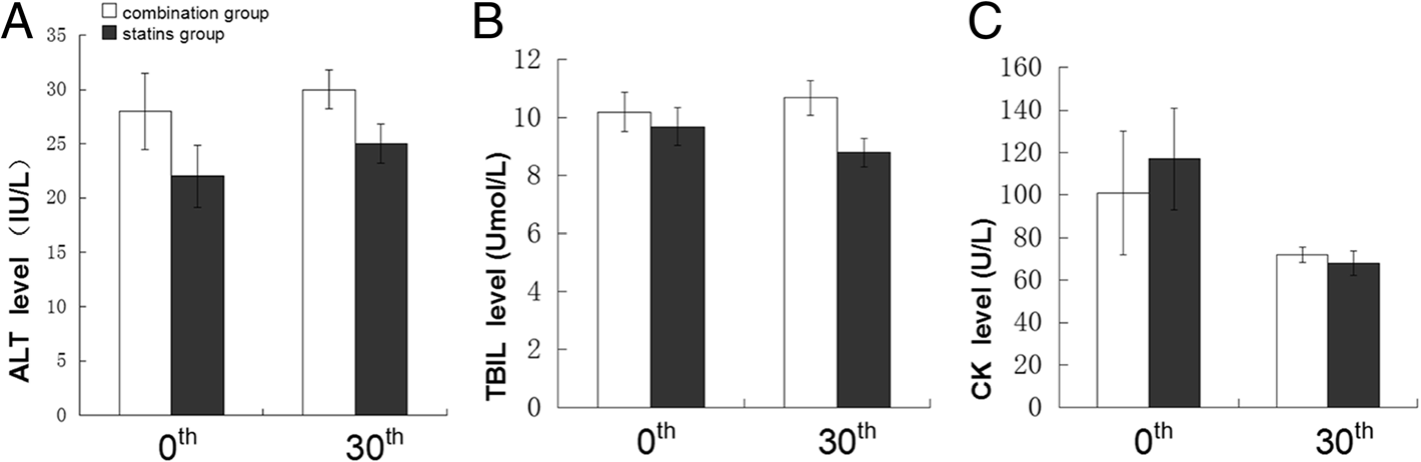
**Safety evaluation during follow-up after treatment.** Liver dysfunction and MRSE marker were from lowest levels within 3 days after admission and at 30^th^ day. **(A, B, C)** liver dysfunction marker ALT, TBIL and MRSE marker CK at 0^th^ and 30^th^ after treatment. Side effects was not observed in combination group and statins group after treatment. Data presented as mean ± SE.

Treatment-related side effects were not observed in this study. No patients showed an increasement in ALT greater than 3 times the ULN or AST greater than 5 times the ULN. No case of hepatitis or jaundice was observed. No patient complained clinical myopathy, and no patient was tested with greater than 5 times elevations of CK levels (Figure 3).

## Discussion

CVD has been demonstrated as one of the most important life-threatening disease in Chinese population [12,13]. Clinical researches verified that the intensive treatment of LDL-C reduced cardiovascular events in patients after MI [14]. The 17% reduction of clinical events was obviously at 30^th^ with intensive lipid-lowering therapy after ACS [15]. Previous study reported that the combination therapy of EZ/40 mg/d Simvastatin produced 52% reduction of LDL-C level in ACS patients [16]. In the present study, we provided the evidence that the treatment of EZ combined with moderate statins can bring 50% reduction of the LDL-C level at 30^th^ in Chinese ACS patients with T2DM. The results demonstrate that the combination group produced more LDL-C reduction during acute phase for Chinese ACS patients with T2DM. The greater reduction of LDL-C level was observed in the combination group compared with statins group (39% VS. 18.5%, p = 0.007) at day 7^th^ after treatment. LDL-C level was reduced by 50% in the combination therapy group at day 30^th^. An additional 21% reduction was observed in patients received combination therapy at day 30^th^. According to the 2011 ESC/EAS guideline for the management of dyslipidemia for patients with very high risk, the target level of LDL-C should be less than 1.8 mmol/L or reduced by 50%, our study show that there are 77% patients in the combination group achieved the recommended LDL-C goal,compared with 45% patients in statins group. This study demonstrated that the combination therapy significantly improve the control rate of LDL-C level during acute phase of ACS in Chinese patients with T2DM. Therefore, adding EZ to moderate statins is more effective strategy to get significant additional LDL-C-lowering and make more Chinese patients to achieve LDL-C goal. Recent study reported that the early reduction of LDL-C level was associated with the reduced cardiovascular events such as recurrent ischemic events and death in ACS patients in the 16 weeks [17]. The present study indicates that stains combined with EZ bring more Chinese ACS patients with T2DM achieve the target LDL-C the goal in early stage, more patients will benefit from lower LDL-C level eventually. Recently, the IMPROVE IT trial demonstrated that the LDL-C level was significantly decreased in EZ/Simvastatin group compared with the simvastatin group in the 30 days. In addition, LDL-C lowering effect in combination therapy continued through-out at least 2.5 years follow-up with good safety and significantly reduced the incidences of CVD morality, MI and stroke.

The levels of TG were decreased by 20% and 22% in combination group and statins group at day 30^th^, respectively, but no significant difference was observed between two groups. However, previous study showed that TG level was decreased significantly in the EZ combined with statins, compared with statins monotherapy [18]. In our study, it is hard to further reduce TG level because the baseline TG level approached to normal level. Our data indicates that the further reduction of TG level produced by EZ is limited when TG level is close to normal.

No improvement on the HDL-C levels were observed in both groups, supporting that either statins or statins combined with EZ were not potent enough to improve HDL-C level in Chinese ACS with T2DM patients.

Previous studies show that the combination of EZ and statins provided greater reduction on hs-CRP compared with statins monotherapy for 12 weeks in hypercholesterolemia patients [19,20]. We observed that the levels of serum hs-CRP were significantly decreased in both groups at day 30^th^, compared with those at baseline. Nevertheless, there was no significant difference between two groups (47% and 42%, P = 0.365) at 30^th^ day. Statins proportion in each group may account for the difference. There were much more rosuvastatin (73% vs. 57%) and less atorvastatin (20% vs. 34%) in statins group, compared with those in the combination group. Moreover, the reduction of hs-CRP levels in each group might be explained by acute inflammation regression after acute stage of ACS and may not be related to the statins or statins combined with EZ. Therefore, based on the present evidence, we cannot overemphasize the anti-inflammatory effects of either statins or EZ [21].

The recent study showed that EZ can improve the IR in the metabolic syndrome with elevated LDL-C (>120 mg/dl) patients in the EZ group [22]. Nonetheless, here we did not observe the effect of EZ on IR in Chinese ACS with T2DM patients. If possible, we would like to further evaluate the effectiveness of EZ on IR in Chinese patients in future study.

Furthermore, we evaluated the safety of combination therapy in the early stage of ACS. No significant elevations of liver enzymes, CK or myopathy were observed in both groups. These data support that the combination therapy of EZ and statins has similar safety as statins monotherapy in Chinese ACS patients with T2DM.

The limitation of our study is small sample size. It is worth to further evaluate the clinical effect of the combined therapy in larger Chinese population of ACS and T2DM with enough longer follow-up.

## Conclusions

In Chinese patients with ACS and T2DM, the combination therapy of EZ and statins at dose used in stable CHD can bring extra LDL-C-lowering effect and improve the control rate of LDL-C level compared with the statins monotherapy. Furthermore, patients with ACS and T2DM should benefit from the early reduction of LDL-C level in combination therapy with safety profile compared with statins group. It is necessary to further investigate the effects and safety of EZ combined with moderate statins in larger Chinese population with ACS and T2DM for longer follow-up, expecting the clinical trial could provide more strong evidence for the application of the EZ and statins in Chinese ACS patients with T2DM.

## Consent

Written informed consent was obtained from the patient for the publication of this report and any accompanying images.

## Abbreviations

CHD: Coronary heart disease
ACS: Acute coronary syndrome
T2DM: Type 2 diabetes mellitus
LDL-C: Low-density lipoprotein cholesterol
CVD: Cardiovascular disease
NSTE-ACS: Non-ST-elevation acute coronary syndrome
EZ: Ezetimibe
NPC1L1: Niemann-Pick C1 like1
IR: Insulin resistance
UA: Unstable angina
NSTEMI: Non-ST-segment elevation myocardial infarction
STEMI: ST-segment elevation myocardial infarction
ULN: Upper limit of normal
CK: Creatine kinase
MRSE: Muscle related side effect
TC: Total cholesterol
TG: Triglyceride
HDL-C: High-density lipoprotein cholesterol
ALT: Alanine aminotransferase
AST: Aspartate aminotransferase
ROS: Rosuvastatin
ATOR: Atorvastatin
SIM: Simvastatin
PRA: Pravastatin
BMI: Body mass index
TBIL: Total bilirubin
Cr: Creatine
FBG: Fasting blood glucose
HbA1C: Glycosylated hemoglobin A1C
TnI: Troponin I
